# Interobserver agreement between eight observers using IOTA simple rules and O-RADS lexicon descriptors for adnexal masses

**DOI:** 10.1007/s00261-022-03580-8

**Published:** 2022-06-28

**Authors:** Neha Antil, Preethi R. Raghu, Luyao Shen, Thodsawit Tiyarattanachai, Edwina M. Chang, Craig W. K. Ferguson, Amanzo A. Ho, Amelie M. Lutz, Aladin J. Mariano, L. Nayeli Morimoto, Aya Kamaya

**Affiliations:** 1 Department of Radiology, Stanford Hospital and Clinics, Stanford, CA USA; 2 Department of Radiology, University of CA – San Francisco, San Francisco, CA USA; 3 Department of Radiology, Santa Clara Valley Medical Center, San Jose, CA USA; 4 Department of Radiology, University of Alberta Hostpial, Edmonton, Alberta Canada

**Keywords:** IOTA, O-RADS, Ovarian, Adnexal, Ultrasound, Pelvic

## Abstract

**Purpose:**

To evaluate interobserver agreement in assigning imaging features and classifying adnexal masses using the IOTA simple rules versus O-RADS lexicon and identify causes of discrepancy.

**Methods:**

Pelvic ultrasound (US) examinations in 114 women with 118 adnexal masses were evaluated by eight radiologists blinded to the final diagnosis (4 attendings and 4 fellows) using IOTA simple rules and O-RADS lexicon. Each feature category was analyzed for interobserver agreement using intraclass correlation coefficient (ICC) for ordinal variables and free marginal kappa for nominal variables. The two-tailed significance level (a) was set at 0.05.

**Results:**

For IOTA simple rules, interobserver agreement was almost perfect for three malignant lesion categories (M2-4) and substantial for the remaining two (M1, M5) with k-values of 0.80–0.82 and 0.68–0.69, respectively. Interobserver agreement was almost perfect for two benign feature categories (B2, B3), substantial for two (B4, B5) and moderate for one (B1) with k-values of 0.81–0.90, 0.69–0.70 and 0.60, respectively. For O-RADS, interobserver agreement was almost perfect for two out of ten feature categories (ascites and peritoneal nodules) with k-values of 0.89 and 0.97. Interobserver agreement ranged from fair to substantial for the remaining eight feature categories with k-values of 0.39–0.61. Fellows and attendings had ICC values of 0.725 and 0.517, respectively.

**Conclusion:**

O-RADS had variable interobserver agreement with overall good agreement. IOTA simple rules had more uniform interobserver agreement with overall excellent agreement. Greater reader experience did not improve interobserver agreement with O-RADS.

**Graphical abstract:**

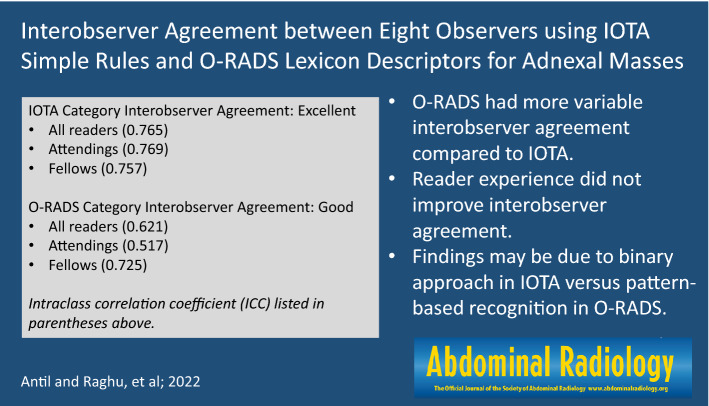

## Introduction

Numerous ultrasound (US) guidelines have attempted to guide the accurate characterization and subsequent management of adnexal masses. These include the International Ovarian Tumor Analysis (IOTA) simple rules, American College of Radiology Ovarian-Adnexal Reporting and Data System (O-RADS), Society of Radiologists in Ultrasound (SRU) Consensus Guidelines, Gynecologic Imaging Reporting and Data System (GI-RADS), and Morphology Index by the University of Kentucky. These systems rely on subjective assessment, pattern-based recognition, morphologic indexing, simple scoring systems, and/or statistically derived algorithms [1–9]. We aim to evaluate interobserver agreement in classification of adnexal masses using two of the most widely used systems: the pre-existing IOTA Simple Rules and newer O-RADS lexicon.

In 2008, the IOTA group published evidence-based nomenclature which led to development of the “Simple Rules” [1, 2]. These include a set of five US features indicative of benignity (B-rules) and a set of five US features indicative of malignancy (M-rules). Based on these rules, adnexal masses are then categorized into benign, malignant, or inconclusive [1, 2]. The system has high diagnostic performance and good risk prediction capability, but still has limited use in clinical practice given the need for further imaging workup of all IOTA inconclusive lesions, which account for approximately 20% of patient cases in one study [9].

In 2019, the American College of Radiology introduced O-RADS to provide an internationally standardized risk-stratified lexicon and to unify various diagnostic and management approaches into a single model [8, 9]. The lexicon provides descriptors and definitions for physiologic cysts (i.e. follicle, corpus luteum) as well as non-physiologic benign and malignant adnexal masses. Based on the lesion descriptors, the system further classifies into six risk categories. These include O-RADS 0, an incomplete evaluation; O-RADS 1, healthy normal premenopausal ovaries or physiological simple cysts ≤ 3 cm; O-RADS 2, almost certainly benign with < 1% risk of malignancy; O-RADS 3, lesions with low (1–10%) risk of malignancy; O-RADS 4, lesions with intermediate (10- < 50%) risk of malignancy; and O-RADS 5, lesions with high (≥ 50%) risk of malignancy [8, 9]. Management or follow-up recommendations are also provided for each category as part of O-RADS.

## Methods

### Study design

A retrospective reader-based diagnostic performance study was performed in women who presented to the radiology department for routine non-obstetric pelvic ultrasound. The research study was Health Insurance Portability and Accountability Act (HIPAA) compliant and received Institutional Review Board (IRB) approval. Due to the retrospective nature of the study, informed consent was waived. A medical record review and review of US images was performed on all women who underwent a routine non-obstetric pelvic US between January 2008 and December 2014 at Stanford University Medical Center, which yielded a total of 7359 exams.

### Inclusion and exclusion criteria

Pelvic ultrasound examinations were reviewed on the picture archiving and communication systems (PACS) workstation by a research radiologist (NA) with specialization and expertise in pelvic ultrasound and ovarian cancer. All exams with adnexal masses (cystic, solid, or mixed cystic and solid) were included in the study. Patients with bilateral adnexal masses were recorded separately as two lesions. Normal or incomplete studies – i.e. without transvaginal scanning or color Doppler – were excluded. Additionally, the following exams were excluded: extra-ovarian lesions, physiologic follicles or corpus luteum, and cystic lesions < 1 cm in post-menopausal women.

**Fig. 1:**
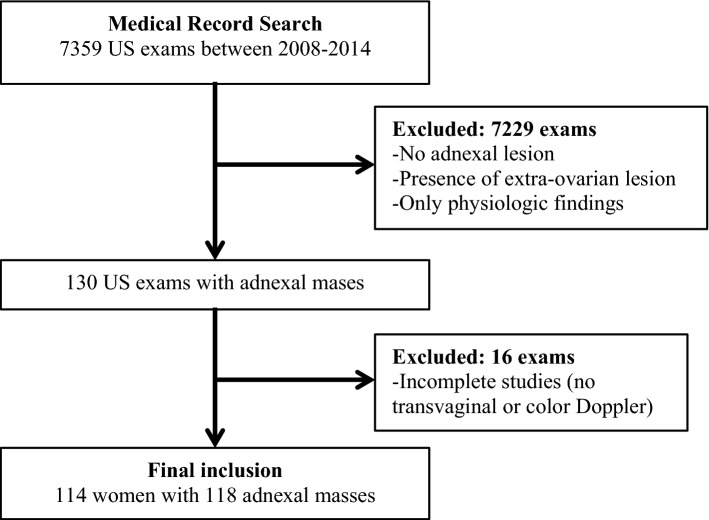
Flowchart on deriving the final cohort

The research radiologist (NA) reviewed the electronic medical records and recorded patient age, menopausal status, and final pathologic diagnosis when available. For lesions that were not resected, adnexal masses with adequate follow-up (≥ 2 years of follow-up with documented imaging to show benignity of the lesion) were included in the final analysis. Imaging follow-up for 2 years on any modality was accepted: ultrasound, computed tomography (CT) or magnetic resonance imaging (MRI) to document stability or resolution. In certain cases, follow-up CT or MRI which characterized a classic lesion was also noted (e.g. macroscopic fat seen on CT or MRI to confirm suspected dermoid cyst). In cases where the imaging comparison was not available in our system, clinician notes indicating stability for 2 years were used in lieu of 2-year imaging follow-up. All data were collected and recorded, with final inclusion of 114 patients with 118 adnexal masses (Fig. 1).

### Image review and data collection

**Fig. 2:**
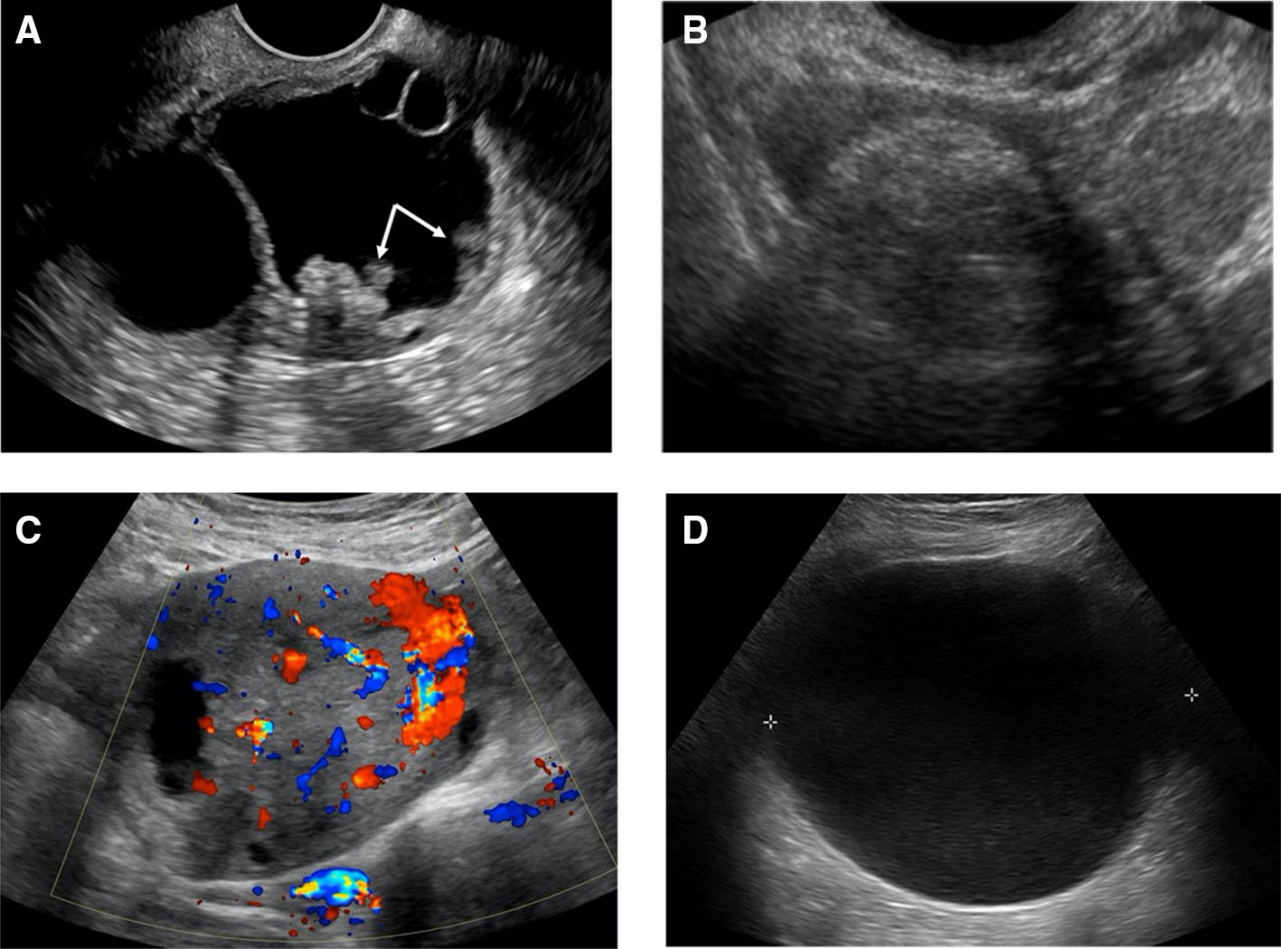
Ultrasound Examples of IOTA Features. **A** Grayscale image of an ovarian multilocular cystic lesion with multiple (≥ 4) papillary projections (white arrows). This is an example of M3: ≥ 4 papillary projections, which had almost perfect interobserver agreement. **B** Grayscale image of an ovarian echogenic lesion with acoustic shadows (example of B3), which had almost perfect interobserver agreement. **C** Color Doppler image of an ovarian solid lesion with very strong blood flow (example of M5), which had substantial interobserver agreement. **D** Grayscale image of an ovarian unilocular cyst (example of B1), which had moderate interobserver agreement

**Fig. 3:**
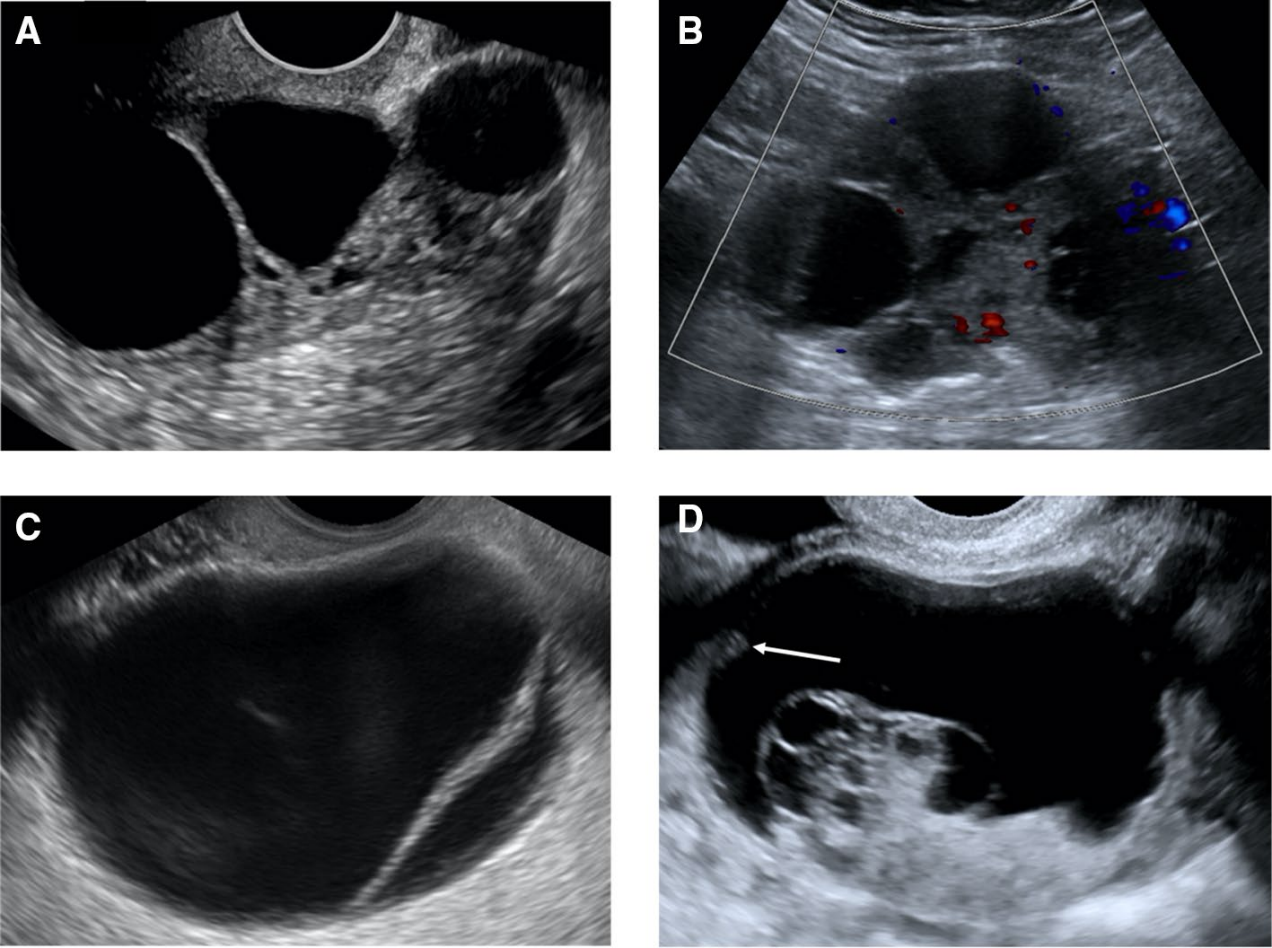
Ultrasound Examples of O-RADS Lexicon. **A** Grayscale image of an ovarian cystic lesion with solid component. This is an example of lesion type, which had substantial interobserver agreement. **B** Color Doppler image of an ovarian cystic lesion with solid component. This is an example of color score 2 (mild), which had good interobserver agreement. **C** Grayscale image of ovarian cyst with a smooth septation. This is an example of septation type, which had moderate interobserver agreement. **D** Grayscale image of a complex ovarian cystic lesion with white arrow denoting the irregular inner wall. This is an example of inner wall, which had fair interobserver agreement

All pelvic US images of included subjects were then evaluated on PACS by eight radiologists at different levels of clinical expertise: 4 fellows and 4 attendings (with 4 years, 4 years, 7 years, and > 15 years of experience). All readers were blinded to the final diagnosis and provided identical training materials on the classification systems. Each adnexal mass was evaluated according to the feature categories of the IOTA Simple Rules (Table 1) and O-RADS lexicon (Table 2). The lesion was then classified into IOTA benign (≥ 1 B-Feature is present with no M-Feature), malignant (≥ 1 M-feature is present with no B-Feature) or inconclusive (both M and B- Features are present or neither is present), with case examples shown in Fig. 2 [2]. Similarly, an O-RADS category (O-RADS 2- 5) is assigned using the O-RADS US risk stratification and management system, with case examples shown in Fig. 3 [9].

**Table 1:** IOTA simple rules

Rules for predicting malignant tumor (M-rules)	Rules for predicting benign tumor (B-rules)
M1: Irregular solid tumor	B1: Unilocular cyst
M2: Presence of ascites	B2: Presence of solid component < 7 mm
M3: At least 4 papillary projections	B3: Presence of acoustic shadows
M4: Irregular multilocular solid tumor with largest diameter ≥ 10 cm	B4: Smooth multilocular tumor with the largest diameter < 10 cm
M5: Very strong blood flow	B5: No blood flow

**Table 2:** O-RADS lesion descriptors

O-RADS features	Categories
Lesion type	1: Simple cyst, 2: Classic hemorrhagic cyst, 3: Classic endometrioma, 4: Classic dermoid, 5: Multilocular cyst without solid component (≥ 1 septa), 6: Cyst with internal solid component (papillary projection), 7: Nodule or large solid component, 8: Mostly solid (> 80%), 9: Unilocular cyst with irregular wall
Inner wall	Smooth, irregular, n/a
Septation type	None, smooth, irregular
Number of septa (< 3 mm)	0, 1, ≥ 2
Number of solid components (> 3 mm)	0, 1, 2, 3, ≥ 4
Contour of solid component	Smooth, irregular, n/a
Color score	1 (absent flow), 2, 3, 4
Ascites & peritoneal implants	No (not present), yes (present)
O-RADS score	0, 1, 2, 3, 4, 5

### Statistical analysis

Each feature category under O-RADS and IOTA was analyzed for interobserver agreement. O-RADS, IOTA, and their subcomponents can be considered as ordinal or nominal variables. For ordinal variables, intraclass correlation coefficient (ICC) was calculated by *ICC* command (*IRR* package) in R Studio version 1.3.1073. We used two-way random-effects model, absolute agreement, single rater type, as previously suggested by a guideline [10]. ICCs were interpreted as follows: < 0.40, poor; 0.40–0.59, fair; 0.60–0.74, good; and 0.75–1.00, excellent [11]. For nominal variables, Free-marginal kappa was calculated using an online calculator (http://justusrandolph.net/kappa) [12]. Free-marginal multirater kappa is an alternative to Fleiss’ multirater kappa. Calculation of chance agreement in Fleiss’ kappa is based on *fixed* marginal probabilities. Thus, Fleiss’ kappa is suitable when raters know beforehand the *fixed* proportions of cases in each category. However, in our study, the raters were blinded to the numbers of cases in each category [12]. Free-marginal kappa is the suitable statistics in our setting. Free-marginal kappa values were interpreted as follows: < 0, poor; 0.01 – 0.20, slight; 0.21 – 0.40, fair; 0.41 – 0.60, moderate; 0.61 – 0.80, substantial; and 0.81 – 1.00, almost perfect agreement [13]. ICCs and kappas, along with 95% confidence intervals (CI), were calculated for agreement amongst all 8 radiologists (4 attendings and 4 fellows). Agreement was also calculated amongst attendings alone and fellows alone. The two-tailed significance level ($\alpha$) was set at 0.05.

## Results

### Subjects and demographics

A total of 114 women with 118 adnexal masses were included in the study, with inclusion and exclusion criteria summarized in Fig. 1. Median patient age was 41 years, with range 18 to 88 years. Age and menstrual status for the cohort, including for benign versus malignant cases, is detailed in Table 3. The median (IQR) lesion size was 7.0 (6.3) cm. All 118 adnexal masses in our study were included as a small subset of a separate and unrelated multi-institutional study evaluating the diagnostic accuracy of O-RADS [25].

**Table 3:** Patient demographics and clinical characteristics

	All Lesions *N* = 118	Benign Lesions *N* = 91 (77%)	Malignant Lesions *N* = 27 (23%)
Age: median (IQR)	41 (32–53)	39 (31–48)	51 (42–64)
Menstrual status			
Pre-menopausal	83 (70%)	72 (79%)	11 (41%)
Post-menopausal	35 (30%)	19 (21%)	16 (59%)
Reference standard			
Surgical pathology	77 (65%)	50 (55%)	27 (100%)
Imaging	41 (35%)	41 (45%)	0 (0%)

Values in pretences are percentage (rounded to whole number) or interquartile range (IQR)

### Imaging or pathologic follow-up

Of the 118 adnexal masses, 77% (91/118) were benign and 23% (27/118) were malignant. Of the benign cases, 55% (50/91) lesions were resected with benign pathology results, whereas 45% (41/91) were not resected and remained stable or decreased in size by imaging or clinical follow-up. Unresected adnexal masses were either stable or decreased in size by imaging for 2 years (ultrasound, CT, or MRI) or deemed stable on clinical notes for 2 years if imaging was not available in our system. Overall, 65% (77/118) of the adnexal masses went on to be resected (Table 3).

### Interobserver agreement

The percentages of O-RADS 0, 1, 2, 3, 4 and 5 categories assigned in this study were 1.1%, 0.1%, 27.5%, 16.2%, 29.6% and 25.5%, respectively amongst all readers. The percentages of IOTA Benign, Inconclusive, and Malignant categories were 55.6%, 17.7% and 26.7%, respectively amongst all readers.

IOTA: Interobserver agreement was almost perfect for three of five malignant lesion categories (M2-4) and substantial for the remaining two malignant feature categories (M1 & M5) of the IOTA simple rules with k-value of 0.80–0.82 and 0.68–0.69, respectively. Interobserver agreement was almost perfect for two of five benign feature categories (B2 & B3), substantial for two (B4 & B5) and moderate for the remaining one benign feature (B1) with k-value of 0.81–0.90, 0.69–0.70 and 0.60, respectively. The final IOTA conclusion was excellent for all eight readers combined, fellows alone, and attendings alone, with ICC values of 0.765, 0.757, and 0.769, respectively (Table 4).

**Table 4:** Interobserver agreement of IOTA simple rules

IOTA features	k*/ICC	All readers	Attendings	Fellows	Discrepancy attendings vs. fellows
M1: irregular solid tumor	k	0.69 Substantial	0.58 Moderate	0.77 Substantial	Yes
M2: ascites	k	0.89 Almost perfect	0.90 Almost perfect	0.88 Almost perfect	No
M3: ≥ 4 papillary projections	k	0.83 Almost perfect	0.80 Substantial	0.85 Almost perfect	Yes
M4: irregular multilocular solid tumor ≥ 10 cm	k	0.83 Almost perfect	0.83 Almost perfect	0.80 Almost perfect	No
M5: very strong blood flow	k	0.68 Substantial	0.64 Substantial	0.69 Substantial	No
B1: unilocular cyst	k	0.60 Moderate	0.59 Moderate	0.64 Substantial	Yes
B2: solid component < 7 mm	k	0.81 Almost prefect	0.75 Substantial	0.88 Almost perfect	Yes
B3: acoustic shadows	k	0.90 Almost perfect	0.88 Almost perfect	0.91 Almost perfect	No
B4: smooth multilocular tumor < 10 cm	k	0.70 Substantial	0.68 Substantial	0.73 Substantial	No
B5: no blood flow	k	0.69 Substantial	0.71 Substantial	0.66 Substantial	No
Final category	ICC	0.765 Excellent	0.769 Excellent	0.757 Excellent	No

*Free-marginal kappa is only calculated to the hundredth by the calculator

O-RADS: Interobserver agreement was almost perfect for two of ten feature categories (presence of ascites and peritoneal nodules) with k-value of 0.89 & 0.97. Agreement in interpretation for remaining eight feature categories (lesion type, inner wall type, septation type, number of septa, number of solid components, contour of solid component and color score) were variable ranging from fair to substantial with ICC and k-value ranging from 0.39–0.61. The final O-RADS conclusion was good for all eight readers combined, good for fellows alone, and fair for attendings alone with ICC values of 0.621, 0.725 and 0.517, respectively (Table 5).

**Table 5:** Interobserver agreement of ACR O-RADS Lexicon

O-RADS Descriptors	k*/ICC	All readers	Attendings	Fellows	Discrepancy attendings vs. fellows
Lesion type	k	0.61 Substantial	0.62 Substantial	0.59 Moderate	Yes
Inner wall	k	0.39 Fair	0.38 Fair	0.39 Fair	No
Septation type	k	0.59 Moderate	0.54 Moderate	0.64 Substantial	Yes
Number of septa	ICC	0.659 Good	0.607 Good	0.698 Good	No
Number of solid components	ICC	0.515 Fair	0.615 Good	0.464 Fair	Yes
Contour of solid component	k	0.55 Moderate	0.48 Moderate	0.56 Moderate	No
Color score	ICC	0.648 Good	0.698 Good	0.639 Good	No
Ascites	k	0.89 Almost perfect	0.90 Almost perfect	0.87 Almost perfect	No
Peritoneal implants	k	0.97 Almost perfect	0.97 Almost perfect	0.98 Almost perfect	No
O-RADS score	ICC	0.621 Good	0.517 Fair	0.725 Good	Yes

*Free-marginal kappa is only calculated to the hundredth by the calculator

## Discussion

Proper characterization and risk stratification of adnexal masses is important because ovarian cancer is the most lethal of all gynecologic malignancies and is the fifth leading cause of cancer-related deaths in women, with an overall 5-year survival rate of only 46% [14, 15]. Ultrasound is the first line initial imaging modality utilized to evaluate the adnexa and to help differentiate benign from malignant ovarian lesions. Multiple ultrasound-based guidelines and scoring systems have been proposed and validated over the years [1–9, 16, 17]. Of these, IOTA simple rules and O-RADS have gained significant traction. We found that interobserver agreement is overall excellent for IOTA simple rules and good for O-RADS.

We hypothesize that the differences in interobserver agreement between the two systems relates to the risk stratification method: while pattern recognition is important in the initial assessment of IOTA simple rules, final delineation into benign, inconclusive, or malignant is based on an algorithmic scoring system. On the other hand, O-RADS is an entirely pattern-based scoring system with potential for some degree of subjectivity and measurement error that may influence this nuanced pattern recognition. We found two features in particular had lower interobserver agreement in O-RADS (scored as “fair”) primarily due to differences in distinguishing smooth versus irregular inner wall and number of solid components. A focus of nodularity along the inner wall may be interpreted as an irregular inner wall by some, whereas others may interpret this finding as a solid component. Measurement differences may further contribute to differences in O-RADS score, as less than 3 mm in size is considered an irregular inner wall whereas 3 mm or greater is considered a solid component. Subjectivity in color Doppler scoring of vascularity can further impact agreement. For example, a solid-appearing mass with color score 2–3 (mild to moderate flow) would be O-RADS 4, whereas a color score 4 would upgrade the mass to O-RADS 5. Finally, some variability in interpretation of what constitutes ‘solid component’ under the O-RADS lexicon may lead to differences in categorization (i.e. fat, Rokitansky nodule, normal ovary within a peritoneal inclusion cyst, tubal or inflammatory tissue in a tubo-ovarian abscess, etc.).

In a study by Basha et al., the diagnostic performance of O-RADS was compared to IOTA and GI-RADS (gynecologic imaging reporting and data system). They found greater sensitivity and similar specificity and reliability with O-RADS compared to the other two [18, 19]. They also found interobserver agreement to be similar across all three risk stratification systems. Although their study had a larger sample of adnexal masses, they used only 5 radiologists, all of whom had greater than 15 years of experience with pelvic imaging and were not blinded to the initial ultrasound reports. A smaller study by Pi et al. [20] with 3 readers and 50 adnexal masses found excellent diagnostic accuracy and interobserver agreement for the O-RADS system but did not compare O-RADS against other existing classification systems.

There have been a few performance comparison studies based on different US scoring systems but none have specifically focused on interobserver agreement. Hiett et al. [22] compared IOTA Simple Rules, ADNEX model, and O-RADS, and stated similar sensitivity for discrimination of malignant from benign pelvic masses with superior specificity with the IOTA model. Patel-Lippman et al. [21] performed a comparison study between IOTA Simple Rules and SRU and demonstrated IOTA Simple Rules slightly more accurate than the SRU guidelines (AUC, 0.9805 versus 0.9713; *p* = 0.0003) and both to be highly sensitive for detection of malignancy. Another study by Xie et al. [23] noted that the area under the curve, sensitivity, and specificity for detection of malignancy under IOTA or O-RADS can be similarly improved by factoring in the patient’s CA-125 levels, as is done in the ADNEX model. Although tumor marker information was not provided to our readers and is often not prospectively available at the time of initial ultrasound interpretation, it certainly plays a role for the gynecologists and gynecologic oncologists in determining management for indeterminate adnexal masses [24].

We included body imaging fellows and attending radiologists with varying years of experience to determine if experience may affect degree of interobserver agreement. All participants were given identical training materials and resources to review beforehand. Interestingly, interobserver agreement amongst both fellows and attendings was excellent for the IOTA simple rules. However, better interobserver agreement was seen amongst the fellows with O-RADS compared to attendings. When analyzing the specific O-RADS and IOTA feature categories, attendings had greater agreement for two O-RADS features (lesion type and number of solid components) compared to fellows. On the other hand, fellows had greater agreement on the other eight features within O-RADS. Thus, number of years of experience did not appear to improve interobserver agreement with O-RADS. It is important to recognize that greater interobserver agreement does not necessarily correlate with diagnostic accuracy. Thus, while fellows may have had greater uniformity than attendings, we caution any inference regarding the diagnostic accuracy of the two groups.

We acknowledge several limitations in our study. First, this was a single institution study. Future larger scale multicenter studies with multiple readers may be warranted to evaluate institutional or regional variability in interpretation and interobserver agreement. Second, we did not evaluate the diagnostic accuracy of each risk stratification system. Thus, while IOTA may have greater interobserver agreement, we do not know if the diagnostic performance of one is better than the other. Indeed, diagnostic performance is of vital importance in determining the merits of each system. Third, images were retrospectively reviewed and therefore may not replicate real-life evaluation of adnexal masses as we were limited to images obtained at the time of examination. As technology and sonographic detail improve, the diagnostic interpretation and accuracy may similarly change. Finally, we did not evaluate the diagnostic performance or accuracy of these two systems when compared to final pathology. Due to the large number of radiologists interpreting each exam, we did not ask radiologists to come to consensus, nor did we determine a “correct” categorization for each ovarian lesion. A separate multi-institutional study with a larger cohort has analyzed the category-specific diagnostic performance of O-RADS [25].

In summary, we found excellent interobserver agreement with IOTA and good interobserver agreement with O-RADS amongst eight blinded observers reviewing 118 adnexal masses. Greater reader experience did not improve interobserver agreement with O-RADS.
